# Preventive effect of permethrin-impregnated long-lasting insecticidal nets on the blood feeding of three major pyrethroid-resistant malaria vectors in western Kenya

**DOI:** 10.1186/1756-3305-7-383

**Published:** 2014-08-20

**Authors:** Hitoshi Kawada, Kazunori Ohashi, Gabriel O Dida, George Sonye, Sammy M Njenga, Charles Mwandawiro, Noboru Minakawa

**Affiliations:** Department of Vector Ecology & Environment, Institute of Tropical Medicine, Nagasaki University, Nagasaki, Japan; Health and Crop Sciences Research Laboratory, Sumitomo Chemical Co Ltd, Hyogo, Japan; School of Public Health, Maseno University, Kisumu, Kenya; Springs of Hope, Mbita, Kenya; Eastern and Southern Africa Centre of International Parasite Control, Nairobi, Kenya; Kenya Medical Research Institute, Nairobi, Kenya; The Global Center of Excellence Program, Nagasaki University, Nagasaki, Japan

**Keywords:** Permethrin, Resistance, *Anopheles gambiae* s.s, *Anopheles arabiensis*, *Anopheles funestus*, LLIN, Kenya

## Abstract

**Background:**

Since the World Health Organization (WHO) adopted the use of long-lasting insecticidal nets (LLINs) as a principal strategy for effective malaria prevention and control, pyrethroids have been the only class of insecticides used for LLINs. The dramatic success of insecticide-treated nets (ITNs) and LLINs in African countries, however, has been threatened by the rapid development of pyrethroid resistance in vector mosquitoes. ITNs and LLINs are still used as effective self-protection measures, but there have been few studies on the effectiveness of ITNs and LLINs in areas where vector mosquitoes are pyrethroid-resistant.

**Methods:**

To investigate the behavioral pattern of mosquitoes in the houses where LLINs were used, indoor mosquito trappings of *Anopheles gambiae* s.s., *An. arabiensis*, and *An. funestus* s.s. were performed with Centers for Disease Control and Prevention (CDC) miniature light trap equipped with a collection bottle rotator at 2-hour intervals between 4:00 pm and 8:00 am. The trapped female mosquitoes were identified and classified as unfed, blood fed, and gravid. The abdominal contents of fed female mosquitoes were used for DNA extractions to identify the blood source.

**Results:**

A large proportion of human blood feeding of *An. arabiensis* and *An. funestus* s.s. (but not *An. gambiae* s.s.) took place during the time people were active outside LLINs. However, during the hours when people were beneath LLINs, these provided protective efficacy as indicated by reduced human blood feeding rates.

**Conclusion:**

LLINs provided effective protection against pyrethroid-resistant malaria vector populations during bedtime hours. However, protection of LLINs was insufficient during the hours when people were active outside of the bed nets. Such limitation of LLINs will need to be intensively addressed in African countries in the near future.

## Background

One of the most successful breakthroughs in the development of pesticides of natural origin has been the discovery of pyrethrum and the successful development of synthetic pyrethroids. Pyrethroids are the predominant insecticides and used in various formulations for mosquito control. Globally, pyrethroids comprise 40% of the insecticides used annually for indoor residual spraying against malaria vectors [1]. The use of ITNs as a simple and inexpensive self-protection measure against malaria has reduced the morbidity of children (<5 years old) by 50% and global child mortality by 20%–30% [2, 3]. However, ITNs required impregnation and reimpregnation with pyrethroids [4], thus the use of LLINs, in which insecticides are incorporated into synthetic fibers and have long residual effects, might be preferable [5]. Since the WHO adopted the use of LLINs as a principal strategy for effective malaria control in the Roll Back Malaria Partnership [6], pyrethroid has been the only class of insecticide used for LLINs [1, 7].

Pyrethroids are safe for mammals and have unique modes of action such as fast knockdown and excito-repellent effects [8]. Although the use of untreated bed nets was already common in some tropical and subtropical countries before the introduction of ITNs [9], several studies have demonstrated the greater effectiveness of ITNs compared with untreated nets [10–13]. Five deltamethrin-coated (or -incorporated) nets, 4 alpha-cypermethrin-coated (or -incorporated) nets, and 2 permethrin-incorporated nets have been listed as WHO-recommended LLINs.

The dramatic success of ITNs and LLINs in African countries, however, has been countered by the rapid development of pyrethroid resistance in vector mosquitoes over the past decade [14]. ITN coverage might be one of the major factors causing such resistance [15], as well as agricultural usage of pyrethroids, and mainly attributable to the fact that malaria vector control currently depends on a single class of insecticides (pyrethroids) [14].

ITNs and LLINs are still used as effective self-protection measures, but there have been few studies on the effectiveness of ITNs and LLINs in areas where vector mosquitoes are pyrethroid-resistant. Personal protection of the two LLINs (Olyset^®^ Net and Permanet^®^) was evaluated in a rice-growing area of south-western Burkina Faso, where *kdr*-governed pyrethroid resistance in *An. gambiae* s.s. was reported, and significantly lower numbers of mosquitoes were collected in LLIN-intervention houses compared to the control houses, indicating the additional deterrent effect of the LLINs [16]. Significantly higher mortality of *An. epiroticus* Linton & Harbach was observed with Permanet^®^ 2.0 and 3.0 despite the fact that the mosquito population showed metabolic resistance to pyrethroids [17]. The aim of this study was to investigate the difference in the reaction of vector mosquitoes to pyrethroid-impregnated bed nets in the area where multi-modal pyrethroid resistance has developed, and the effect of LLINs on these malaria vectors is discussed.

## Methods

### Study site

The study area was the Mbita and Suba districts of Nyanza Province in western Kenya. The rainfall pattern in the area is bimodal, with the long rainy season occurring from March to May and the short rainy season occurring in November and December. Malaria infection rates rise steadily between September and February and peak briefly in June, following the long rains [18]. The Mbita and Suba districts have been identified as high vector transmission areas in Kenya, and more than 50% of the population is exposed to malaria at a rate of ≥40% *Pf* PR_2–0_ (*Plasmodium falciparum* parasite rate corrected to a standard age range of 2 to <10 y) [19]. Kawada *et al*. [20, 21] reported that the 3 major malaria vectors, *Anopheles gambiae* Giles (*An. gambiae* sensu stricto [s.s.]), *An. arabiensis* Patton, and *An. funestus* Giles (*An. funestus* s.s.), in the study area were highly resistant to pyrethroids and that resistance mechanisms were multimodal, including the knockdown resistance (*kdr)* caused by the point mutation of the voltage-gated sodium channel (L1014S) in *An. gambiae* s.s. and cytochrome P450–related metabolic factors in both *An. arabiensis* and *An. funestus* s.s. Recently Kawada *et al*. [22] also reported the insecticide resistance status of the three malaria vectors in the present study area. *Anopheles gambiae* s.s., *An. arabiensis*, and *An. funestus* s.s. showed high resistance to both permethrin and deltamethrin. The KT_50_s of the 3 species for permethrin and deltamethrin were >60 minutes except for deltamethrin in *An. arabiensis* (KT_50_, 29.5 minutes), indicating low knockdown activities of the 2 pyrethroids against these mosquitoes as well as low killing activities. In contrast, lethal activities of fenitrothion and propoxur against the three species were higher (95–100% mortality) than those of the 2 pyrethroids.

### Indoor collection of mosquitoes by miniature light traps equipped with collection bottle rotators in the houses using LLIN

**Figure 1:**
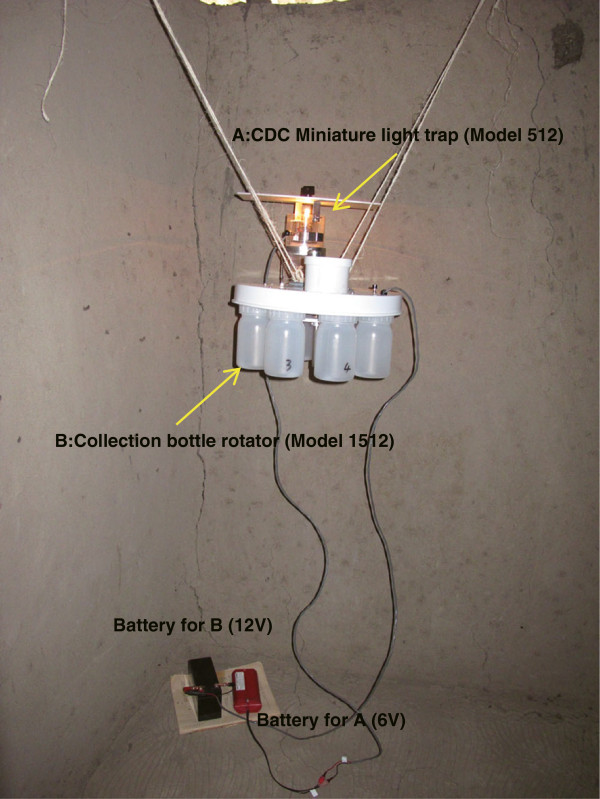
**Photo of the CDC miniature light trap (model 512) equipped with a collection bottle rotator (model 1512) for all-night mosquito collection at 2-hour intervals (4:00** **pm** **to 8:00** **am** **).** The trap was hung from the eaves with string and placed at a height of approximately 1.2 m in a corner of the living room away from the place where people slept.

**Figure 2:**
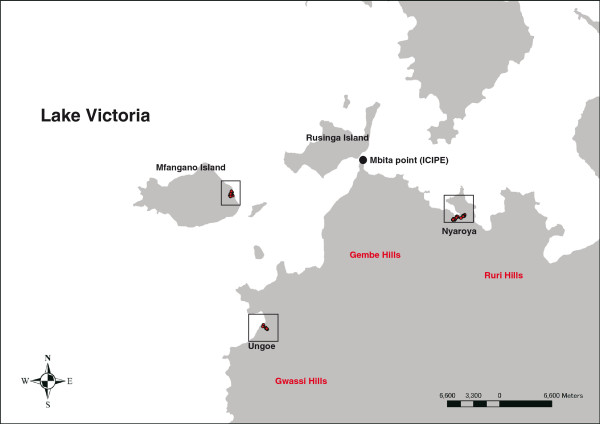
**Map of the sites for mosquito collection.** Red circles indicate houses used for mosquito collection.

To investigate the behavioral pattern of mosquitoes, indoor mosquito trapping of *An. gambiae* s.s., *An. arabiensis* and *An. funestus* s.s. was performed with 6 sets of the CDC miniature light trap (model 512) equipped with a collection bottle rotator (model 1512) (John W. Hock Co., FL, USA) (Figure 1). Indoor trapping of *An. gambiae* s.s. was performed in 6 houses on Mfangano Island from May 16 to June 1, 2011. Indoor trapping of *An. arabiensis* and *An. funestus* s.s. was conducted in 6 houses in Nyaroya Village from October 4 to 31, 2011, and July 11 to 18, 2012, and in 6 houses in Ungoe Village from February 21 to March 6, 2012 (Figure 2). The residents of the houses were informed about the study, and their written consent was obtained before mosquito collection. The houses used for indoor mosquito collection were of standard construction for the area; they had mud walls and eaves, traditional opening structures between the roof and the walls, and had 1–2 bedrooms and a living room. Before the start of the study, each house was inspected and untreated bed nets and LLINs were exchanged for new permethrin-incorporated LLINs (Olyset^®^ Nets; Sumitomo Chemical Co., Ltd., Tokyo, Japan). Additional new Olyset^®^ Nets were provided for use in the living room when the residents (most were children >5 years) slept in the living room with no bed nets. Tables 1 and 2 show the number of Olyset^®^ Nets, the number of persons who slept in the bedroom(s) and living room, and the approximate number of livestock for each house.

**Table 1:** **Information on the houses used for all-night trapping of** ***An. gambiae*** **s.s. by CDC traps equipped with a collection bottle rotator in May to June 2011**

House no. ^a^	No. of olyset ^®^nets	No. of persons sleeping		No. of livestock around house		
		Bedroom ^b^	Living room	Cow	Sheep	Goat
MFA 1	3	2A	3C	13	0	7
MFA 7	2	2A	3C	7	30	10
MFA 9	2	1A + 1C	0	5	0	0
MFA 11	1	1A + 1C	0	7	30	10
MFA 12	2	1A	2A	3	0	0

^a)^MFA, Mfangano; A, adults (>15 years old); C, children (≤15 years old).

^b)^Persons sleeping in a bedroom usually used one bed under a single bed net.

**Table 2:** **Information on the houses used for all-night trapping of** ***An. arabiensis*** **and** ***An. funestus*** **s.s. by CDC traps equipped with a collection bottle rotator**

House no. ^a^	Date of collection	No. of olyset ^®^nets	No. of persons sleeping ^b^		No. of livestock around house		
			Bedroom ^c^	Living room	Cow	Sheep	Goat
NYAR 11	Oct 2011 Jul 2012	2^d^	2A	1C	18	2	26
NYAR 14	Oct 2011 Jul 2012	1	1A	0	22	8	9
NYAR 15	Oct 2011 Jul 2012	2^d^	2A	0	0	0	2
NYAR 19	Oct 2011 Jul 2012	2	2A	0	2	0	0
NYAR 20	Oct 2011 Jul 2012	2	2A + 3C	0	22	8	9
NYAR 21	Oct 2011	1	1A	0	2	0	0
NGO 3	Feb–Mar 2012	2	2A	1C	8	0	10
NGO 4	Feb–Mar 2012	1	2A	0	6	0	4
NGO 5	Feb–Mar 2012	2	1A	2C	10	0	4
NGO 6	Feb–Mar 2012	2	2A	1C	10	0	4
NGO 7	Feb–Mar 2012	3	2A	4C	0	0	0
NGO 8	Feb–Mar 2012	2	1A + 2C	0	10	0	4

^a)^NYAR, Nyaroya; NGO, Ungoe.

^b)^A, adults (>15 years old); C, children (≤15 years old).

^c)^Persons sleeping in a bedroom usually used one bed under a single bed net.

^d)^One PermaNet^®^ 2.0 was used occasionally instead of an Olyset^®^ Net in the living room.

The collection bottle rotator was programmed to collect active mosquitoes at 2-hour intervals between 4:00 pm and 8:00 am into 8 different plastic bottles. The trap was hung from the ceiling with strings and placed at a height of approximately 1.2 m in a corner of the living room away from where people slept. The trapped female mosquitoes were identified and classified as unfed, blood fed, and gravid. The abdominal contents of fed female mosquitoes were used for DNA extractions to identify the blood source. One to 6 replicates in each house (total of 21 replicates) on Mfangano Island, 2 to 16 replicates in each house (total of 43 replicates) in Nyaroya Village, and 2 to 15 replicates in each house (total of 88 replicates) in Nyaroya and Ungoe villages were performed for collection of *An. gambiae* s.s., *An. arabiensis*, and *An. funestus* s.s., respectively.

### Species identification

Adult mosquitoes were examined microscopically to distinguish *An. gambiae* s.l. and *An. funestus* s.l. from other anophelines based on identification keys developed by Gillies and Coetzee [23]. Multiplex polymerase chain reaction (PCR) methods described by Scott *et al*. [24] and Koekemoer *et al*. [25] were used for the species identification.

### Identification of meal sources in blood-Fed mosquitoes

Mosquitoes collected in the field were individually placed in 1.5-mL plastic tubes containing silica gel desiccant and stored at -10°C until processed. Engorged abdomens were separated from the rest of the body for blood source identification. DNA was extracted from the blood in each abdomen using the REDExtract-N-Amp Tissue PCR Kit (Sigma, MO, USA) per the manufacturer’s instructions. Species identification of the host blood source was performed by multiplex PCR as described by Kent and Norris [26] using published cytochrome B primers for human (human741F, GGCTTACTTCTCTTCATTCTCTCCT) and cow (cow121F, CATCGGCACAAATTTAGTCG) and a universal reverse primer (UNREV1025, GGTTGTCCTCCAATTCATGTTA). A 20-μL cocktail consisting of 0.5 μmol/L of each primer and previously diluted (300 times) 1 μL of the DNA template was placed in a 0.2-mL cell containing AccuPower™ PCR Premix (Bioneer, Daejeon, Korea). PCR was performed under the following conditions: a hot start at 95°C for 5 min; 35 cycles of template denaturing at 95°C for 1 min, primer annealing at 54°C for 1 min, and amplicon extension at 72°C for 1 min; and a final extension at 72°C for 7 min.

### Detection of point mutations in the voltage-gated sodium channel

PCR and direct DNA sequencing were performed to identify point mutations at 1014 L in the field-collected mosquitoes according to the method by Kawada *et al*. [20, 21]. Direct DNA sequencing was performed using the 3730 DNA Analyzer (Applied Biosystems). The electropherogram of the targeted amino acid replacement was analyzed using MEGA 4.0 public domain software ( http://www.megasoftware.net/). The unique DNA haplotype sequences were deposited into GenBank.

### Data analysis

A digital map in shape file format (Kenya-Boundaries, FAO Africover, http://www.glcn.org/activities/africover_en.jsp) was used for mapping of the collection sites. The geographical positions of the collection sites were plotted on the map using ArcGIS 10.1 (ESRI Japan Corp, Tokyo, Japan).

The daily average number of mosquitoes collected per house in the 8 time zones was calculated. The generalized linear mixed model using the poisson distribution was used to examine whether the collection times explained the activity patterns using the package lme4 in R. The house and the date of collection were used as random effects. The multiple comparison of the number of collected mosquitoes at different time zones was performed by means of Tukey honestly significant difference test using the package multcomp in R. Comparison of the number of human blood–fed and unfed mosquitoes during bedtime (10:00 pm–6:00 am) and active time (4:00–10:00 pm and 6:00–8:00 am) was performed with χ^2^ test using JMP 7.0 J (SAS Institute Japan Inc., Tokyo, Japan).

### Ethics statement

The protocol for the study (case no. 1775) was approved by the Scientific Steering Committee and the National Ethics Review Committee of the Kenya Medical Research Institute. All necessary permits were obtained for the described field studies. No mosquito collection was done without the approval of the head of the village and the owner and occupants of the collection house.

## Results

**Figure 3:**
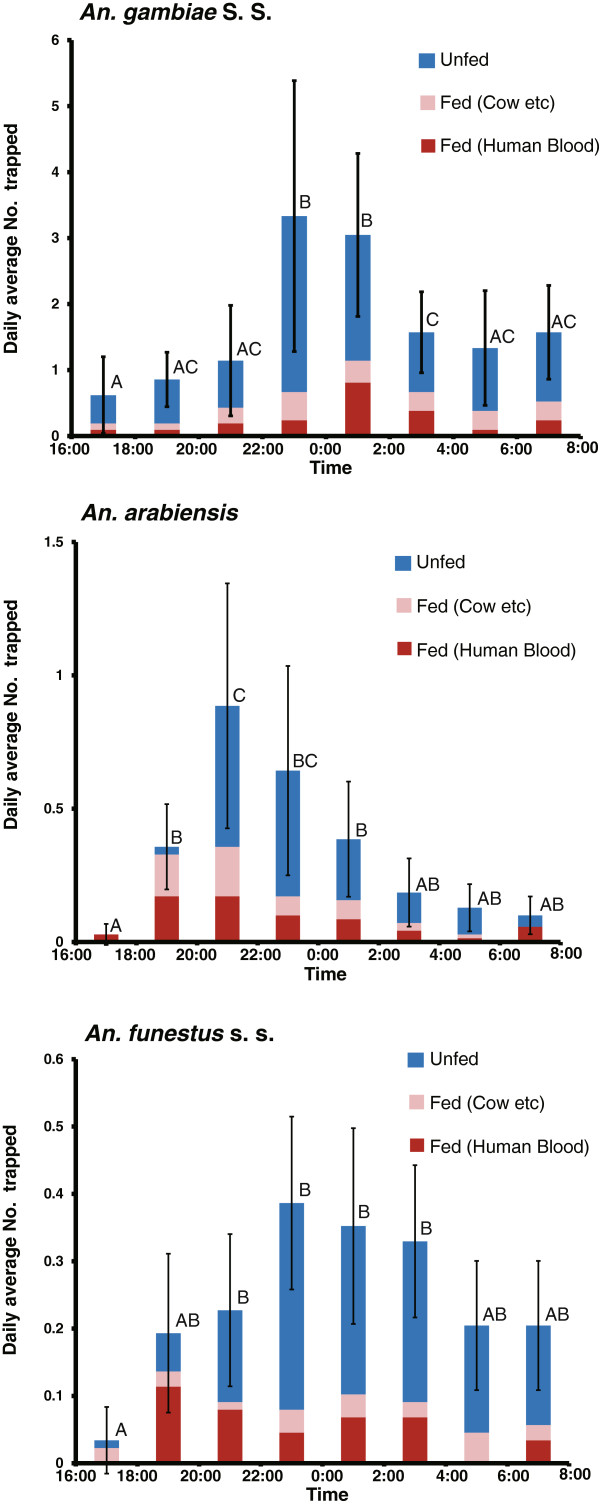
**Indoor activity pattern of** ***An. gambiae*** **s.s.,** ***An. arabiensis*** **, and** ***An. funestus*** **s.s. female mosquitoes trapped by CDC miniature traps equipped with a collection bottle rotator in houses in which LLINs were used.** Different letters indicate the significant difference (Tukey honestly significant difference test, p < 0.05), and bars indicate the 95% confidence limits for the total number of mosquitoes collected at each time period.

**Figure 4:**
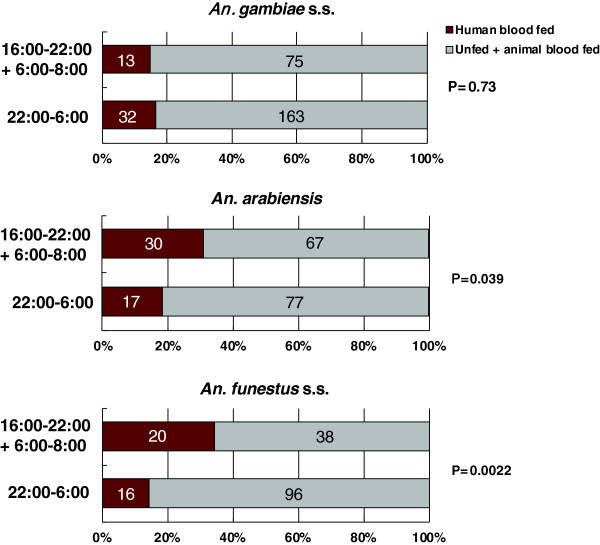
**Number of female mosquitoes collected at bedtime (10:00 PM to 6:00 AM) and active time (4:00–10:00 PM and 6:00–8:00 AM) using a CDC miniature trap equipped with a collection bottle rotator.**

The indoor activity patterns of the 3 major malaria vectors in western Kenya are shown in Figure 3. The number of trapped female *An. gambiae* s.s. (fed and unfed) significantly rose at night (10:00 pm to 2:00 am), falling before and after this period (ANOVA, df = 7, χ^2^ = 84.9, p < 0.0001). The number of human blood–fed mosquitoes also peaked during this period (ANOVA, df = 7, χ^2^ = 26.7, p < 0.001), but there was no significant peak in the number of animal blood–fed mosquitoes (ANOVA, df = 7, χ^2^ = 7.20, p = 0.41). The allelic frequency of the point mutation in the voltage-gated sodium channel (L1014S) in the collected mosquitoes was 98.4% (n = 96, Accession AB776705, AB776706). Among the 283 female *An. gambiae* s.s. collected (13.4 ± 0.70/house per a night), 195 were unfed (68.9%, 9.3 ± 0.81/house per a night), 45 contained blood from a human source (15.9%, 2.0 ± 0.26/house per a night), and 43 had fed from an animal source (15.2%, 2.0 ± 0.26/house per a night). Among the 45 female mosquitoes engorged with human blood, 22 (48.9%) were trapped from 10:00 pm to 2:00 am. There was no significant difference in the ratio of human blood–fed mosquitoes to unfed plus animal blood–fed mosquitoes between bedtime (10:00 pm to 6:00 am), when most residents were assumed to sleep under bed nets, and active time (4:00–10:00 pm and 6:00–8:00 am), when most residents were active outside bed nets (χ^2^ = 0.03, p = 0.73, Figure 4).

The number of trapped female *An. arabiensis* (fed and unfed) significantly rose from early night (8:00–10:00 pm) to midnight (10:00 pm to 12:00 am), falling before and after this period (ANOVA, df = 7, χ^2^ = 122.6, p < 0.0001) (Figure 3). The number of human blood–fed (ANOVA, df = 7, χ^2^ = 22.0, p = 0.0025) and animal blood–fed mosquitoes (ANOVA, df = 7, χ^2^ = 37.5, p < 0.0001) peaked earlier than the above period (6:00–10:00 pm). Among the 191 female *An. arabiensis* collected (2.7 ± 0.32/house per a night), 106 were unfed (55.5%, 1.5 ± 0.32/house per a night), 47 contained blood from a human source (24.6%, 0.67 ± 0.19/house per a night), and 37 had fed from an animal source (19.4%, 0.53 ± 0.13/house per a night). Among the 47 female mosquitoes engorged with human blood, 24 (51.1%) were trapped from 6:00 to 10:00 pm. A significant difference in the ratio of human blood–fed mosquitoes to unfed plus animal blood–fed mosquitoes was observed between bedtime (10:00 pm to 6:00 am) and active time (4:00–10:00 pm and 6:00–8:00 am) (χ^2^ = 3.58, p = 0.039, Figure 4).

The number of trapped female *An. funestus* s.s. (fed and unfed) showed broader peaks as compared to those of *An. gambiae* s.s. and *An. arabiensis*, with significant peaks through almost the whole night (8:00 pm to 4:00 am) (ANOVA, df = 7, χ^2^ = 39.7, p < 0.0001) (Figure 3). The number of human blood–fed mosquitoes also showed broad peaks that peaked earlier at night (6:00–10:00 pm) (ANOVA, df = 7, χ^2^ = 25.7, p < 0.0001), whereas there was no significant peak in the number of animal blood–fed mosquitoes (ANOVA, df = 7, χ^2^ = 2.49, p = 0.93). Among the 170 female *An. funestus* s.s. collected (1.9 ± 0.12/house per a night), 115 were unfed (67.6%, 1.3 ± 0.14/house per a night), 36 contained blood from a human source (21.2%, 0.41 ± 0.038/house per a night), and 19 had fed from an animal source (11.2%, 0.22 ± 0.12/house per a night). Among the 36 female mosquitoes engorged with human blood, 17 (47.2%) were trapped from 6:00 to 10:00 pm. A significant difference in the ratio of human blood–fed mosquitoes to unfed plus animal blood–fed mosquitoes was observed between bedtime (10:00 pm to 6:00 am) and active time (4:00–10:00 pm and 6:00–8:00 am) (χ^2^ = 8.17, p = 0.0022, Figure 4).

## Discussion

In Nyanza province, dieldrin was reported to be administered mainly through aerial spraying especially for tsetse fly control through 1968–1971 [27], while the organized intensive spray of dichloro-diphenyl-trichloroethane (DDT) for mosquito control was not performed in the 1970s and 1980s, and no indoor residual spraying (IRS) has been administered since then. Therefore, the extensive use of ITNs and LLINs in the study area is thought to be a major factor causing high pyrethroid resistance [21, 28].

In the present report we hypothesized that activity patterns of mosquitoes as indicated by the number trapped in the light trap corresponded to the host seeking or blood feeding activity patterns. The hypothesis is strongly supported by the experimental data of Kawada and Takagi [29] who showed that the activity patterns recorded by the photoelectric sensing devises in the laboratory well explained the host seeking activity patterns in the field as indicated by the number of mosquitoes by human bait collection. The activity of *An. gambiae* s.s. peaked at midnight (10:00 pm–2:00 am), and human biting corresponded to this activity pattern. There was no significant difference in the ratio of human blood–fed female mosquitoes to unfed + animal blood–fed female mosquitoes between bedtime (10:00 pm–6:00 am) and active time (4:00–10:00 pm and 6:00–8:00 am), suggesting that LLINs did not affect human blood feeding activity of *An. gambiae* s.s., although the LLINs reduced the feeding success in this species at low level (15.9%). The similar consistency of anthropophagy in *An. gambiae* s.s. under the existence of permethrin-impregnated bed nets was reported by Bøgh *et al*. [30]. The activity peak in *An. arabiensis* was early at night (10:00 pm–12:00 am), followed by human and animal biting peaks (6:00–10:00 pm), and human biting at bedtime was significantly reduced as compared with active time in this species. The small human biting peak was also noted in the morning (6:00–8:00 am), when most people were assumed to be active outside of bed nets. In comparison to the 2 previously described species, the activity peaks in *An. funestus* s.s. had a broader range throughout the night. However, the same discrepancy in active time and human blood feeding time as shown in *An. arabiensis* was observed in this species. Additionally, the small human biting peaks were noted in the morning (6:00–8:00 am) in this species.

In conclusion, LLINs were effective against 3 major malaria vectors during bedtime, since the percentages of human feeding success were found to be reduced to a low level during bedtime (15.9–24.6%). Although we could not arrange the untreated control houses in the present study simply because of ethical reason, feeding successes of *An. gambiae* in the houses without LLINs or with insecticide-untreated and badly torn bed nets were reported to be more than 80% [31, 32]. The overall activities of the 3 major malaria vectors in this study area have not changed from those reported before the extensive use of ITNs and LLINs started in Africa [33–35]. However, a notable human blood feeding activity in evening and a slight activity in early morning was shown in both *An. arabiensis* and *An. funestus* s.s., while there was no such events in *An. gambiae* s.s. The same human biting patterns were recently reported in *An. gambiae* s.l. (most of which were thought to be *An. arabiensis*) [36], *An. arabiensis* [37], and *An. funestus* s.s. [36, 38]. Though simple explanation for such species dependent difference in the host feeding activities under LLIN use observed in the present report is difficult, the difference in physiological factors such as the intrinsic host preference, insecticide resistance mechanisms, and repellency to pyrethroids, etc. might be involved as the plausible answers. Gatton *et al*. [39] recently reviewed the behavioral changes in mosquitoes by the impact of indoor insecticides. Most interventions against malaria vectors have involved indoor treatment of insecticides such as IRS and ITNs and they are thought to have selected exophagy or exophily and time shift of human feeding. Bayoh *et al*. [40] reported the historical population decline in *An. gambiae* s.s., but it was not easy to explain this population decline simply because of the changes in climate, the variations in abundance of cattle, etc. The authors concluded that the high coverage of LLINs during the decades was one of the most plausible answer to the above decline. However, the killing effect of LLINs against *An. gambiae* s.s. population, which has developed *kdr* mutations at high frequency (>90%), seemed to be not enough to cause such population decline. The historical population decline of this species might also be explained by the behavioral characteristics in *An. gambiae* s.s. population observed in the present report. *Anopheles gambiae* s.s. might have not changed their characteristics as "midnight feeder" by some physiological reasons notwithstanding existence of LLINs and has to rely on the limited human blood sources, most of which are protected by LLINs, resulting in the decline in their population size.

Bed nets are effective only when the vector mosquitoes are endophagous and their feeding time corresponds to the bedtime. The most important limitation for the effective use of bed nets is that they are only effective when people are sleeping inside. LLINs might not be effective in cases where most blood feeding takes place when people are active outside of bed nets. The importance of the human host feeding in malaria vectors during when peoples are active outside of LLINs as reported in the present study will need to be intensively addressed in African countries in the near future. Changes in house design, such as screening or closing of eaves, might be effective in reducing human exposure to malaria vectors in such conditions [41, 42]. The use of excito-repellency of pyrethroids as a spatial repellent, which acts as a spatial barrier to invasion of mosquitoes or reduces feeding motivation of mosquitoes might be another countermeasure [43].

## Conclusion

LLINs provided effective protection against three pyrethroid-resistant malaria vector populations (*An. gambiae* s.s., *An. arabiensis*, and *An. funestus* s.s.) during bedtime hours. However, protection of LLINs was insufficient during the hours when people were active outside of the bed nets in *An. arabiensis* and *An. funestus* s.s. Screening or closing of eaves and the use of excito-repellency of pyrethroids as a spatial repellent might be effective in reducing human exposure to malaria vectors in such conditions.
